# Micro-/Nano-Texturing onto Plasma-Nitrided Tool Surface by Laser Printing for CNC Imprinting and Piercing

**DOI:** 10.3390/mi13020265

**Published:** 2022-02-06

**Authors:** Tatsuhiko Aizawa, Tomoaki Yoshino, Yohei Suzuki, Tadahiko Inohara

**Affiliations:** 1 Surface Engineering Design Laboratory, SIT, Tokyo 144-0045, Japan; 2 Komatsu-Seiki Kosakusho, Co., Ltd., Suwa 392-0012, Japan; yoshino@komatsuseiki.co.jp (T.Y.); y-suzuki@komatsuseiki.co.jp (Y.S.); 3 LPS-Works, Co., Ltd., Tokyo 144-0033, Japan; inohara@lps-works.com

**Keywords:** surface decoration, laser printing, micro-nano texturing, imprinting, piercing

## Abstract

A new data transformation method for micro-manufacturing using a topological model for a micro-/nano-texture was proposed for a surface-decorated product. Femtosecond laser printing was utilized to form the micro-/nano-textures into the hardened thick layer of dies by plasma nitriding. At first, the plasma-nitrided AISI316L flat substrate was laser-printed as a punch to imprint the tailored nano-textures onto the AA1060 aluminum plate for its surface decoration with topological emblems. Second, the plasma-nitrided SKD11 cylindrical punch was laser-trimmed to form the nanostructures on its side surface. This nano-texture was imprinted onto the hole surface concurrently with piercing a circular hole into electrical steel sheet. The fully burnished surface had a shiny, metallic quality due to the nano-texturing. The plasma nitriding, the laser printing and the CNC (computer numerical control) imprinting provided a way of transforming the tailored textures on the metallic product.

## 1. Introduction

**Figure 1:**
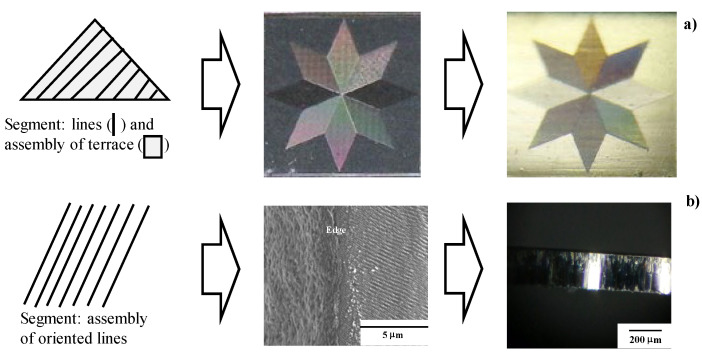
Two data transformation procedures from the design base to the final product by imprinting mother die textures. (**a**) Micro-/nano-patterns → Laser-printed die → Micro-/nano-textured product, and (**b**) nano-patterns → Laser-machined die → Highly qualified product surface.

Digital manufacturing requires innovative changes to the tools used on the production-line [1]. The tailored design geometry must be accurately duplicated on the product surface through data transformation [2]. The product quality must also be improved, even in normal manufacturing, by the tooling design without loss of tool life [3]. In the former, a mother die is utilized as a physical proof to transform the topological geometry of the design onto the micro-/nano-textures on the product surface, as shown in Figure 1a.

In the latter, a mother punch works as a tailored tool to transform a perfect shearing process design onto highly qualified product surfaces with use of the nano-texturing method, as shown in Figure 1b.

Femtosecond laser processing is commonly employed as tooling to perform the laser printing of micro-/nano-textures onto mother dies [4,5]. Among its several features, LIPSS (laser-induced periodic surface structuring) has attracted many researchers since its initial discovery [6]. Through micro-/nano-texturing by femtosecond laser processing, the original hydrophilic surfaces of AISI304 substrates were controlled to be super-hydrophobic [7]; for example, the static contact angle increased to 170.4°, nearly equal to the ideal angle by theoretical estimate [8]. In addition to this surface property control, optical properties were also tuned by femtosecond laser irradiation [9,10]. Hence, the femtosecond laser nano-texturing becomes a key technology to modify and control the physical and chemical properties of various material surfaces in practice [11]. As surveyed in [12], this nano-texturing process is useful to fabricate nano-textured punches and dies for the transcription of the surface nanostructure onto metal, polymer, and oxide-glass products by fine stamping. In addition to this direct printing of nano-structured patterns by stamping, there are many ways to imprint tailored nano-textures onto selective surface areas during metal forming [13]. 

In the present paper, a three-step procedure is proposed to design micro-/nano-textures for the surface decoration of products, to apply tailored textures onto the surface of nitrided punch for micro-embossing and for micro-piercing, respectively, and to imprint them onto product surfaces. These formed nano-textures on the surface of punch are imprinted onto an AA1060 plate surface as well as the pierced hole surface of an electrical steel sheet by using the CNC (computer numerical control) stamper. The product quality is improved by these imprinted micro-/nano-textures.

## 2. Experimental Procedure

### 2.1. Three-Step Procedure from Topological Design to Micro-/Nano-Texuring to Products

**Figure 2:**
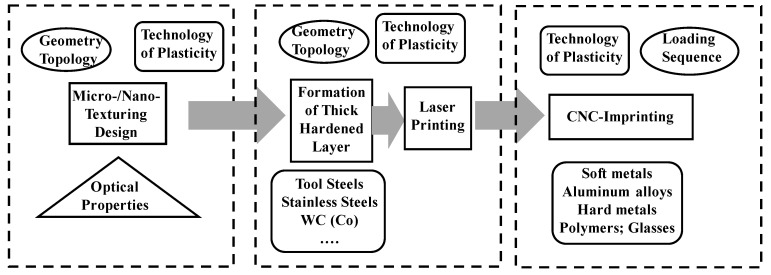
Three-step procedure from the geometric and topological design on the micro-/nano-patterns to its imprinting onto the product surface via tooling.

The three-step procedure is depicted by a scheme in Figure 2. In the micro-/nano-texturing design, a targeting zone is represented by an assembly of micro-textured geometry and nano-textured topology. This CAD (computer-aided design) data are transformed to CAM data for laser printing. In this tooling, the mother die is modified to have a thick, hardened layer with engineering durability under high-stress transients. The original CAD data are transformed to a micro-/nano-textured surface on the laser-printed die and punch surfaces. Various die and punch materials are selected for this laser-printing procedure. 

In the third stage, this laser-printed die is fixed into a die set and loaded to imprint the designed textures onto the product surface. The flow stress of the product materials and the loading sequence in the stamping have a significant influence on the dimensional accuracy of micro-/nano-textures in duplication by stamping. Various product materials are also selected for this imprinting procedure.

### 2.2. Topolical Design for Micro-/Nano-Texturing

**Figure 3:**
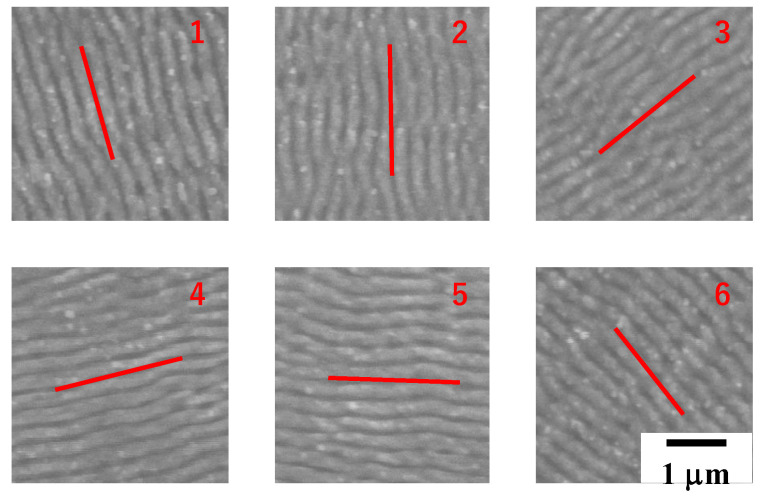
Six nano-textures with different orientations, from #1 to #6, formed onto the stainless steel plates by the optical path control.

Nano-textures induced by LIPSS are controllable through optical path control. Figure 3 depicts how to control the orientation of nano-textures. The width of each LIPSS ripple is mainly determined by the pulse width. As discussed in [12,13], this orientation control of nano-textures reflects on the surface plasmonic diffraction. 

**Figure 4:**
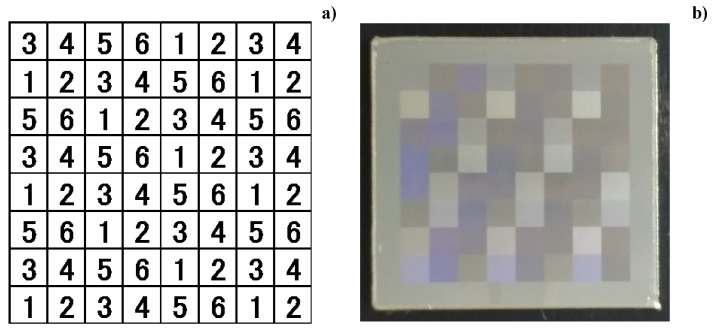
Qualitative control of surface plasmonic state by varying the nano-texture orientation. (**a**) Digital classification of nano-textures for segmentation in laser nano-texturing, and (**b**) nano-textured panel with 8 × 8 segments identified by the different surface-plasmonic reflections.

The six orientations above, in Figure 3, are varied and allotted into each square segmentation on the matrix with eight rows × eight columns, as shown in Figure 4a. Subsequently, the panel nano-textured by this matrix on the AISI316L plate has surface plasmonic colors, as shown in Figure 4b. 

This qualitative correlation between the nano-textures by LIPSS and the surface plasmonic colors is employed to identify the presence of nano-textures on the laser-processed surfaces on the nitrided punch as well as the replica surfaces on the products. Through this nano-texturing together with color-grating though micro-texturing, the laser-printed die surfaces and the imprinted product surfaces develop surface plasmonic brilliance.

### 2.3. Tooling by Nirogen Supersaturation and Laser Printing for Micro-/Nano-Texturing

**Figure 5:**
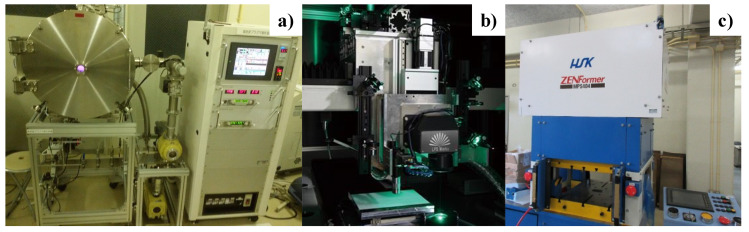
Three systems for tooling and imprinting procedures. (**a**) Low-temperature plasma nitriding system to form the thick hard layer onto the SKD11 punch, (**b**) femtosecond laser machining system to print the micro-/nano-textures into the hardened layer, and (**c**) CNC-stamping system for imprinting these textures onto the product surfaces.

The insertion of a thick, hardened surface layer into the punch and die is necessary to make laser printing with a sufficient depth. The low-temperature plasma nitriding system (PN-03; YS-Electrical Industry, Co., Ltd.; Kohfu, Yamanashi, Japan) in Figure 5a was employed to form a nitrogen supersaturated surface layer without the precipitation of iron and chromium nitrides onto AISI316L and SKD11 punches, respectively. These punches and SKD11 die were plasma-nitrided at 673 K for 14.4 ks by 70 Pa. Other plasma processing parameters were optimized on the basis of the fundamental data analyzed by the plasma diagnosis [14].

The femtosecond laser machining system (Femto-01; LPS-Works, Co., Ltd., Tokyo, Japan) in Figure 5b was utilized for laser-printing the designed micro-/nano-textures. The wave length of the femtosecond laser was 515 nm, the pulse width was 200 fs, and the repetition rate was 20 MHz. The maximum power, on average, was 40 W, and the maximum pulse energy was 50 μJ. Hence, the power by irradiation of a single pulse was estimated to be 0.25 GW. The beam spot diameter was 1 μm.

### 2.4. CNC Stamp Imprinting

The CNC stamping system (Hoden-Seimitsu, Co., Ltd.; Kanagawa, Japan) in Figure 5c was employed to imprint the micro-/nano-patterns on the punch onto the product. The loading sequence was controlled to perform single-step stamping with a constant speed of 0.1 mm/s and to perform multi-step stamping with the loading sequence control.

## 3. Experimental Results

Two types of nitrided punches are prepared for the CNC imprinting of seven micro-textured emblems to the metallic plate and for nano-texturing the metallic sheets with piercing. SEM (scanning electron microscopy)–EDX (electron dispersive X-ray spectroscopy) was utilized to perform the microstructure analysis and nitrogen mapping, respectively.

### 3.1. Preparation of Nitrided Punches

**Figure 6:**
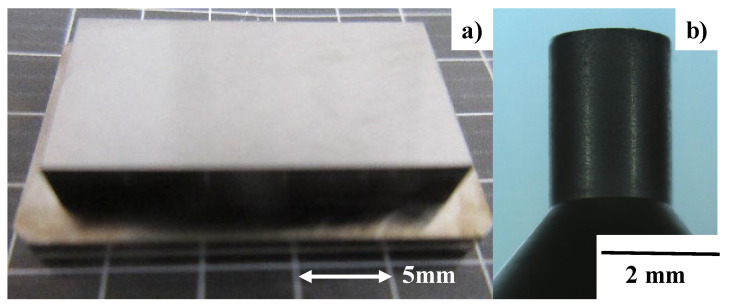
Plasma-nitrided SKD11 punches at 673 K for 14.4 ks. (**a**) Flat punch for CNC imprinting, and (**b**) cylindrical punch for piercing with nano-texturing.

**Figure 7:**
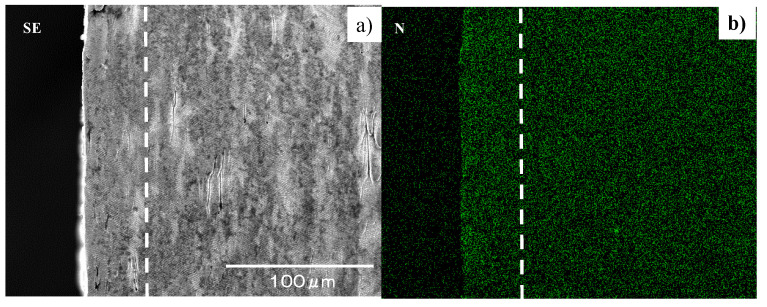
Detailed microstructure and nitrogen mapping of nitrided SKD11 punch. (**a**) SEM image of its cross-section, and (**b**) nitrogen mapping.

High-density RF (radio frequency)–DC (direct current) plasma nitriding was employed to harden and strengthen the AISI316L flat and SKD11 cylindrical punches. Two types of stamping punch were fabricated, as depicted in Figure 6. Both punches have a nitrided layer with a thickness of 50 μm and an average hardness of 1400 HV. Figure 7 depicts a cross-sectional SEM image and nitrogen mapping obtained using the cylindrical punch in Figure 6b.

The average nitrogen content in the nitrided layer reaches 3.4 mass%. Its surface roughness remains as it was before nitriding. The nitrided layer has a fine microstructure, in which the average grain size is reduced to 0.1 μm.

### 3.2. Femtosecond Laser Printing onto Flat Nitrided Punch

These two punches were processed by femtosecond laser printing to form the tailored micro-/nano-textures onto them. The nitrided flat AISI316L punch in Figure 6a was laser-printed to produce seven micro-textured emblems. Each emblem was designed by a polygonal model in geometry, in which each emblem was composed of polygonal segments and each segment was shaped as an assembly of micro-edges and concave micro-terraces in correspondence to the line segments and their spaces in the original 3D CAD data.

**Figure 8:**
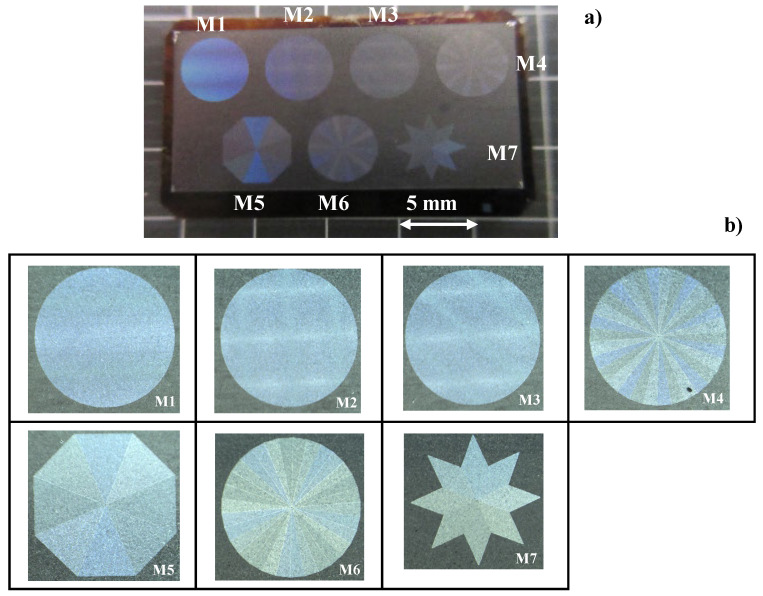
Seven emblems, laser-printed onto the nitrided flat SKD11 punch. (**a**) Overall view of punch surface, and (**b**) optical-microscopy images of seven emblems.

**Figure 9:**
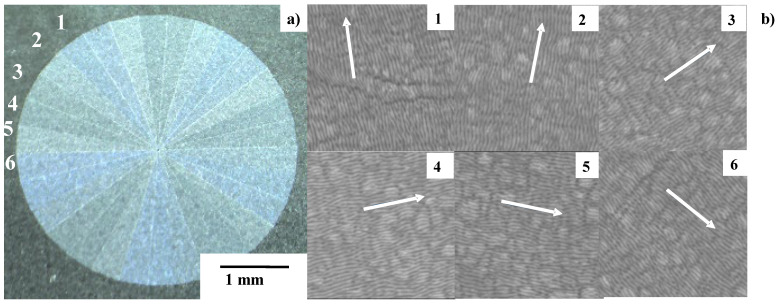
Micro-/nano-texturing in the femtosecond laser printing of M6 onto the nitrided flat punch. (**a**) Construction of M6 by micro-texturing in five repetitions of six segments, and (**b**) nano-texturing with controlled orientation in each segment from #1 to #6 in Figure 8a.

Figure 8a depicts the seven emblems, laser-printed onto the nitrided flat AISI316L punch. These emblems have color-grating by their micro-textures and surface plasmonic brilliance in dark blue by nano-textures. Each emblem in Figure 8b is cut into the nitrided flat punch with the size of 4 mm × 4 mm. The micro-/nano-texture formation in the emblem of M6 is explained in Figure 9.

This M6 emblem is composed of 5 × 6 = 30 triangular segments by repeating the micro-texturing five times process to form six different segments, as depicted in Figure 9a. Every segment in M6 is cut into the nitrided flat punch by using the same polygonal geometry model in CAD. Hence, each triangular segment is micro-textured by alternately aligning the micro-edge and micro-terrace with a distance of 10 μm. In a similar manner to the control of the orientations in the nano-textures in Figure 3, the nano-texture orientation is incrementally rotated 30° anti-clockwise from #1 segment to #6 segment in Figure 9b. The nano-texture width (or LIPSS ripple width) is 300 nm. This procedure to form six anti-clockwise segments is repeated five times to cut the whole M6 emblem onto the flat punch. Many zones with surface angulation are observed on the nano-textured surfaces in Figure 9b. This is due to the local surface roughness, induced by the lattice expansion in the γ-phase AISI316L zones during the nitrogen supersaturation process in the plasma nitriding of AISI316L die at 673 K for 14.4 ks [14]. The average surface roughness is limited, at most, by 0.1 μm. The orientations of the nano-textures in Figure 9b are insensitive to the surface angulation and laser printing with the tailored angle across zone boundaries. This proves that the nitrided punch surface has nothing to do with the LIPSS effect on nano-texturing. In the above laser printing from M1 to M7, every micro-texture is accurately printed into the nitrided layer of punch with much smaller statistical tolerance than the maximum deviation of 2 μm, when using the galvanometer for laser beam control.

### 3.3. Femtosecond Laser Printing onto Nitrided Punch for Piercing

**Figure 10:**
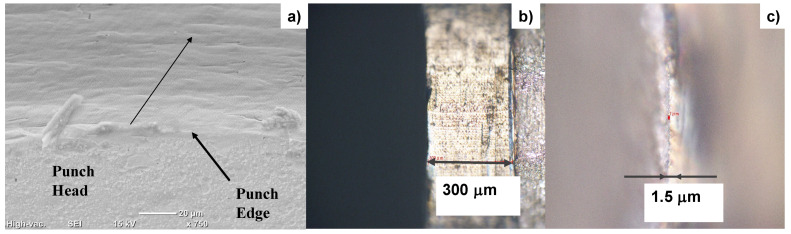
The laser-trimmed SKD11 punch. (**a**) SEM image on the nitrided SKD11 punch after femtosecond laser trimming, (**b**) laser-trimmed side surface of punch, and (**c**) the edge width of trimmed punch.

Both the head and the side surfaces of the plasma-nitrided cylindrical SKD11 punch with a diameter of 2 mm were trimmed by the femtosecond laser processing to sharpen their edges and to reduce the surface roughness. Figure 10a depicts the trimmed top and side surfaces of the nitrided punch across its edge. This punch side surface was laser-trimmed down to a depth of 6 μm along a length of 300 μm, as depicted in Figure 10b. Nano-textured grooves were expected to superpose onto the trimmed surface simultaneously with the femtosecond laser trimming process. Owing to the two-step laser trimming on the punch head and side surfaces, the punch edge was sharpened, as shown in Figure 10c. The original dull edge width of around 10 μm was sharpened down to 1.5 μm by this trimming.

**Figure 11:**
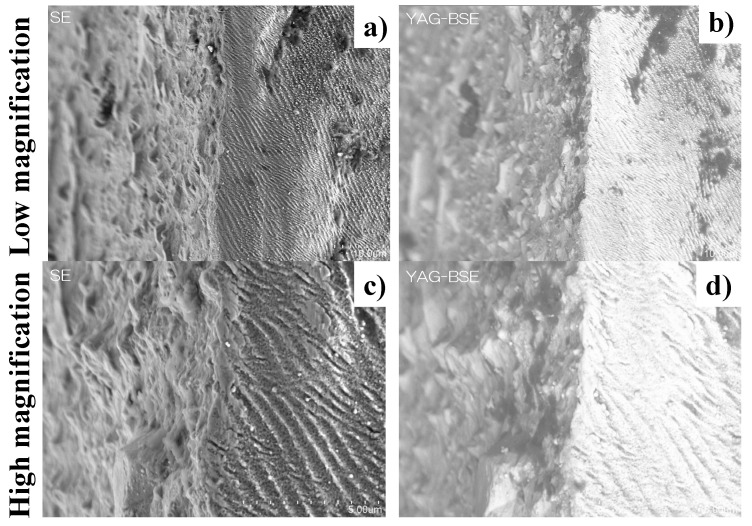
SEM and YAG-BSE images on the top and side surfaces of laser-trimmed SKD11 punch across its edge (**a**,**b**) in low magnification, and (**c**,**d**) in high magnification.

Figure 11 depicts the SEM image on the top and side surfaces of the laser-trimmed SKD11 punch across its edge in low and high magnifications. The nitrided SKD11 punch head is trimmed to produce a smooth surface with an average roughness of less than 0.6 μm. This trimmed top surface changes to the nano-textured side surface across the sharpened edge. This nano-texture orientation is 60°, as tailored for the piercing punch. The LIPSS ripple width also reaches 300 nm in this laser trimming.

### 3.4. CNC Stamp Imprinting to Metallic Products

**Figure 12:**
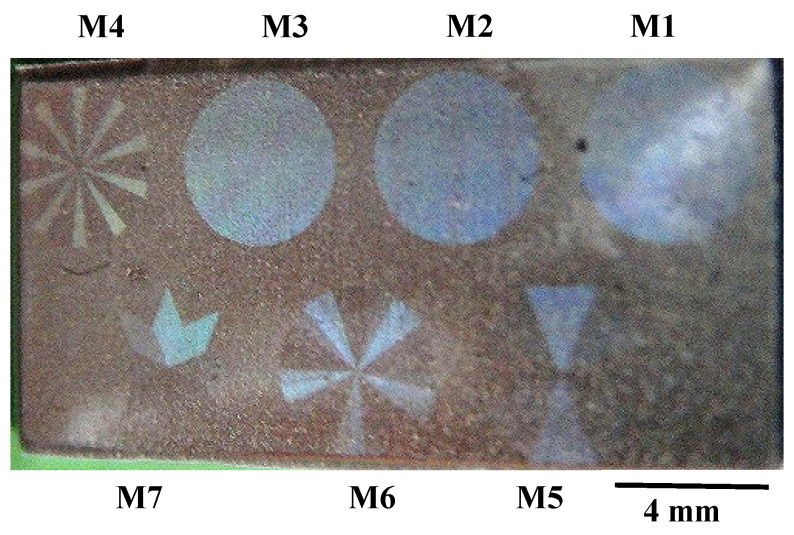
AA1060 pure aluminium plate with the seven micro-textures transcribed by CNC imprinting with the use of the laser-printed AISI316L die in Figure 8 and Figure 9.

An AA1060 aluminum plate with a thickness of 1mm with a normal flow stress of 200 MPa was employed. The CNC stamping system was utilized to imprint seven micro-textures with the tailored nano-textures onto this substrate. A single-mode loading sequence was used to compress the upper punch with a constant velocity. Figure 12 depicts the embossed AA1060 aluminium plate after single-shot loading down to 150 μm from the original position where the punch touched just on the work material surface. This stroke, which was larger than the heights of the micro-textures and nano-textures in Figure 8 and Figure 9, included the elastic response in the support as well as the deformation of rough plate surfaces. Seven micro-textures on the plate also demonstrated surface plasmonic brilliance. This proves that the nano-textures in Figure 8 and Figure 9 also coined into the pure aluminium plate surface.

**Figure 13:**
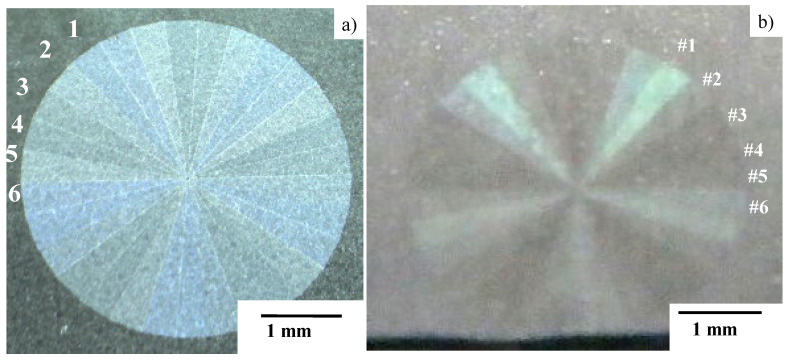
Comparison between the M6 emblem laser-printed onto the AISI316 flat die and the M6-replica, CNC-imprinted onto the AA1060 plate. (**a**) Original M6 emblem on the die, and (**b**) M6-repica on the aluminum plate.

We will now describe the CNC-imprinting behavior of each emblem on the laser-printed die onto the aluminum plate. An M6 emblem was also employed for this comparison. As shown in Figure 13, the original M6 emblem was imprinted onto the aluminum plate in the mirror-image inversion. That is, as shown in Figure 13a, six segments from 1 to 6 in anti-clockwise order in M6 were imprinted to six segments from #1 to #6 in clockwise order in the replica of M6, as shown in Figure 13b. This proves that the original micro-textures on the laser-printed die were accurately coined onto the aluminum plate.

**Figure 14:**
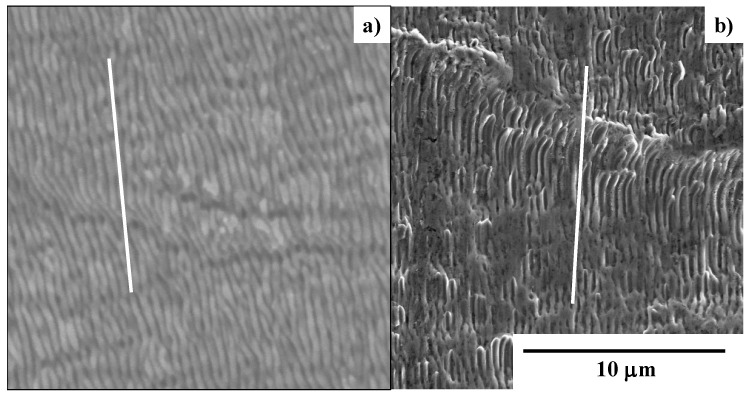
Comparison of SEM images at high magnification of the nano-textures between the 1 segment in the original M6 emblem on the die surface and the #1 segment in the M6 replica on the aluminum plate. (**a**) Original nano-textures, laser-printed on the AISI316L die, and (**b**) replica nano-textures, CNC-imprinted onto the aluminum plate.

An SEM analysis with high magnification was performed to describe the nano-texture imprinting onto the aluminium plate together with the transcription of the micro-texture M6 in Figure 9. Figure 14 compares the SEM images between the original nano-texture in segment #1 in the M6 emblem in Figure 9 and the nano-textures coined onto the aluminum plate surface. Each nano-texture in Figure 14b is aligned in the uniaxial directions with the same ripple width and depth as the laser-printed nano-textures, shown in Figure 14a. The orientations of the nano-textures in Figure 14a,b were in mirror-image inversion; for example, −30° in the original nano-textures in Figure 14a, corresponds to +30° in the coined nano-textures in Figure 14b. This comparison of the nano-textures reveals that the tailored micro-textures in Figure 8 and Figure 9 and nano-textures in Figure 14a were concurrently imprinted accurately onto the work material surface.

### 3.5. Simultaneous Imprinting with Piercing to Metallic Sheets

**Figure 15:**
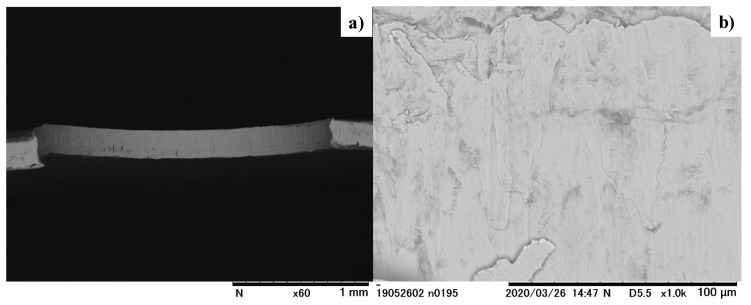
An electrical steel sheet perforated with the use of edge-sharpened and nano-textured SKD 11 punch. (**a**) Overall image of the pierced hole, and (**b**) fully burnished hole surface.

The laser-trimmed SKD11 punch was fixed into the upper die set for piercing the electrical steel sheet with a thickness of 0.15 mm. The core die was also cemented to the lower die set. The CNC stamping system was also utilized for the fine piercing experiment. Figure 15 depicts the electrical steel sheet perforated by this piercing. As shown in Figure 15a, no fractured areas were seen on the pierced hole surface. After the SEM analysis of this pierced hold surface, the whole surface was fully burnished, as shown in Figure 15b. 

We experimentally evaluated the laser-trimmed SKD11 punch life and, here, we describe the debris particle deposition’s behavior. With respect to the former, the recent demand for highly cost-competitive motor cores has resulted in the prolongation of punch life together with the high qualification of the pierced electrical steel sheets. In the latter, chipping and micro-cracking was expected to occur through the severe deposition of debris particles from the work. The original nano-texture was designed to have a straight orientation along the piercing direction in order to visualize the deposition process.

**Figure 16:**
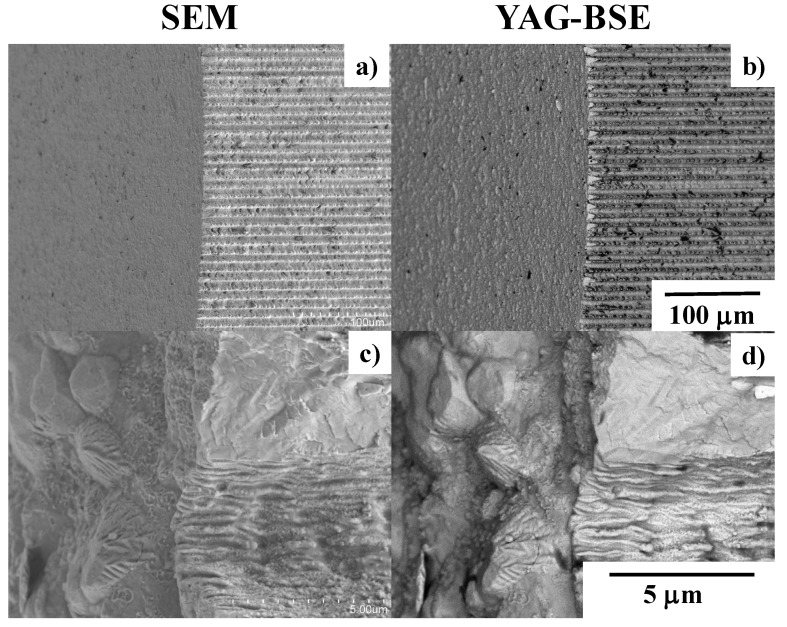
SEM and YAG-BSE images on the top and side punch surfaces across their edges after continuously piercing in 10,000 shots. (**a**,**b**) Low magnification, and (**c**,**d**) high magnification.

Figure 16 depicts the SEM and YAG-BASE images on the top and side surfaces of the laser-trimmed piercing SKD11 punch after punching out the electrical steel sheet with a thickness of 0.15 mm in 10,000 shots. No damage or cracks were observed on the punch edges, shown in Figure 16a,b. The lateral lines denote the debris particle deposition layers. As seen in Figure 16c,d, no particles were deposited onto the bottom of the micro-textures with a pitch of 10 mm. They deposited on the top surface near the edge and on the top of the micro-textures. This proves that almost all debris particles generate, deposit along the micro-textures, and eject themselves from the punch edge to the outside of piercing space. As was the case in [15,16,17], this self-ejection mechanism is useful for preventing the damage induced by the accumulation of debris particles during piercing.

## 4. Discussion

Plasma nitriding, femtosecond laser printing, and the CNC imprinting were employed as a three-step procedure to improve the design flexibility of the micro-/nano-textures of products and to prolong the tool life, in order to ensure high cost-competitiveness. The plasma nitriding process is the first step in the formation of thick, hardened layers into stainless steel and tool steel die, roll and tool substrates. Owing to the low-temperature plasma nitriding, this layer is uniformly nitrogen-supersaturated without nitride precipitates [14]; the die material has sufficient wear and corrosion toughness, as reported in [14,18]. Femtosecond laser printing is the second step to cut any tailored micro-/nano-textures into the material’s surface and interface [15]. Since the whole textures are formed on the nitrided layer, their surfaces have sufficient hardness not to be deformed during the imprinting process of work materials with normal flow stress, such as aluminum and aluminum alloy works. CNC stamping and rolling is the third step used to easily imprint the laser-printed textures onto the product surface with shaping [16]. 

The data transformation from the tailored model in CAD (computer-aided design) to the surface-decorated product by using the three-step procedure becomes a key process to improve the quality of metal and polymer products. The original color grating by micro-texturing and the surface plasmonic brilliance by nano-texturing are duplicated onto the product surface. The colored emblems, symbols, codes, and fonts are embedded into the product surface, as tailored in CAD. In particular, their topology and geometry work as functional convex and concave textures to control the surface properties and to stimulate the engineering performance across the textured interface.

In addition to aluminum and aluminum alloy works, low-flow-stress metals and alloys, such as tin and copper, as well as polymers, such as PET (polyethylene terephthalate) and PMMA (polymethyl methacrylate) can be employed as work materials for cold and warm imprinting. When using the aluminum alloy, the micro-textures are nearly fully imprinted by embossing the tool surface; the nano-textures are often partially transcribed. These metals with low flow stress and polymers are suitable for the full transcription of micro- and nano-textures onto their surfaces.

The fine piercing and punching of metals and alloys requires their sharp separation without the formation of fractured surfaces [19]. As reported in [20], the reduction of clearance between the punch and the die is a normal way to increase the burnished surface–area ratio; the punch and die life is often shortened to lower cost-competitiveness in the production of motor-cores. In the shearing process of electrical steel sheets, the affected zones are inevitably induced during piercing process to increase the iron loss in the motor core [21]. Even under narrowed clearance conditions, the elasto-plastically strained zones by punching distort the magnetic zone structure to increase the hysteresis loss in the motor core [17]. Furthermore, the amorphous electrical steel sheets are at risk of having their magnetic properties significantly deteriorated by these affected zones [22]. 

The present three-step procedure provides a hardened SKD11 punch and die with a sharpened edge and micro-/nano-textured side surfaces. As discovered in [23], the use of a cylindrical punch with a sharp edge and a textured side surface reduces the defects and damages in amorphous steel sheets. In parallel with this high qualification in piercing, the punch life is also prolonged to improve cost-competitiveness and sustainability, or green manufacturing. The fine topological design on the micro-/nano-texturing is necessary to reduce the risk of chipping at the punch and die edges without adhesion wear by debris particles.

## 5. Conclusions

The laser nano-texturing process works directly as a tool to build up a master-piece for the duplication of tailored nano-textures onto metallic and polymer products. Owing to the nitrogen supersaturation of the die substrate at 673 K, the laser-nano-textured die is responsible for long-term usage in this duplication processes. In particular, this nitrided die has sufficient chemical stability and heat resistance to perform high-temperature injection molding and mold stamping for repetitive duplication operations. 

Femtosecond laser nano-texturing also works to form the unidirectional nano-textures onto the trimmed punch side surface. The laser trimming and nano-texturing processes work simultaneously to adjust the surface roughness and geometric irregularities by trimming and to superpose the nano-textures onto the trimmed surface.

## Data Availability

Not applicable.
